# Practical Notes on Diagnosis and Treatment

**Published:** 1909-01-23

**Authors:** 


### Practical Notes on Diagnosis and Treatment.

### Acute Yellow Atrophy of the Liver.

It is important to remember that, although simple catarrhal jaundice is common and acute yellow atrophy very rare, there is no means of distinguishing between the two at the onset. Every case of simple jaundice in a young woman is worth careful watching for this reason.

### The Concomitants of Sea-sickness.

In voyages of only a few hours' duration a profuse diarrhœa is not infrequent, but after a time constipation becomes more marked. The urine is apt to be scanty, and causes smarting in its passage.—*Sir W. H. Allchin.*

### Vaccination.

It is a noteworthy fact that the worst arms are the most septic, not the best vaccinated, and that the most "successful" vaccinators are often the most septic in their operations. The septicity of the arm is no guarantee of the possessor's immunity from smallpox.—*Corner and Pinches.*

### Laparotomy for Tuberculous Peritonitis.

Probably the cases in which surgical interference is most valuable are those in which there is much ascites and some fibro-caseous change in the peritoneum, but the adhesions are not numerous or dense enough to prevent the evacuation of the fluid. The outlook is poor when there is no ascites, but much fibrous change dragging back and binding down the intestine. In such cases but little good is to be expected from laparotomy.—*Dr. Hale White.*

### Carcinoma of the Tongue.

The future of the operative surgery of carcinoma of the tongue undoubtedly lies in early diagnosis of the disease, and in the routine removal of the glands before they are obviously enlarged.—*Mr. Butlin.*

### Scarlatinal Throat.

The strictly scarlatinal factor in the throat is a vivid red injection of the whole of the fauces, palate, and buccal mucous membrane. There may be much, or little if any, swelling or ulceration of the tonsils; the amount is, as it were, purely a matter of accident in respect to the diagnosis of scarlet fever.—*Dr. F. F. Caiger.*

### Pediculosis.

One of the most cleanly and efficient applications is the following lotion:—Hydrarg perchlor. gr. xij., Sp. Vini Rect. ℥ii., Liq. Cocci ʒi. aq. ad ℥vj. Of course this must not be applied when there is any breach of the surface, as so frequently happens in children when the hair of the scalp is affected.

### Smokers and Anæsthesia.

When about to administer a general anæsthetic to a man who admits or is known to be a heavy smoker and has the chronic pharyngitis often associated with such excess, much difficulty from spasm during the induction stage can be avoided by first spraying the throat with a two-per-cent. solution of cocaine. Even better still, though less often practicable, is it to prohibit the habit almost entirely for a week before the intended operation.

